# Suboptimal Use of Effective Contraceptive Methods in Young Mexican Women With Breast Cancer

**DOI:** 10.1200/JGO.18.00064

**Published:** 2018-10-09

**Authors:** Andrea Castro-Sanchez, Bertha Alejandra Martinez-Cannon, Alejandra Platas, Alejandro Mohar, Alan Fonseca, Yoatzin Vega, Adrian Fimbres-Morales, Cynthia Villarreal-Garza

**Affiliations:** **Andrea Castro-Sanchez**, **Bertha Alejandra Martinez-Cannon**, **Alejandra Platas**, **Alejandro Mohar**, **Alan Fonseca**, **Yoatzin Vega**, **Adrian Fimbres-Morales**, and **Cynthia Villarreal-Garza**, Joven y Fuerte: Programa para la Atencion e Investigacion de Mujeres Jovenes con Cancer de Mama; **Andrea Castro-Sanchez**, Instituto Nacional de Cancerologia; **Alejandra Platas**, **Alejandro Mohar**, and **Cynthia Villarreal-Garza**, Instituto Nacional de Cancerologia, Mexico City; and **Bertha Alejandra Martinez-Cannon** and **Cynthia Villarreal-Garza**, Tecnologico de Monterrey, Monterrey, Mexico.

## Abstract

**Purpose:**

Contraceptive counseling and adherence in young women with breast cancer (BC) is a relevant issue because chemotherapy and hormonal treatment resulting in amenorrhea do not preclude unintended pregnancies. Currently, there is limited evidence from high-income countries; however, there are no studies regarding use of contraceptives in patients with BC in Mexico. This study aimed to determine the rate of contraceptive use in young Mexican women with BC during cancer treatment, characterize their contraceptive preferences, and assess contraceptive counseling by Mexican physicians.

**Patients and Methods:**

A cross-sectional survey was conducted regarding contraceptive use and counseling among women age 40 years or younger at BC diagnosis who had completed chemotherapy in the previous 5 years or who were currently receiving long-term treatment with hormonal therapy and/or trastuzumab at a large tertiary health care facility in Mexico.

**Results:**

Of a total of 104 eligible women with median age at diagnosis of 34 years, 51.1% reported using a contraceptive during chemotherapy and 45.7% reported using a contraceptive during other types of cancer treatment (hormonal therapy and trastuzumab). Of the 51 patients (49%) who were sexually active during chemotherapy, 76.5% used contraception, but only 29.4% used an effective contraceptive method. When asked about contraceptive counseling, only 16.7% recalled being advised by their health care provider. Sexually active women who received contraceptive counseling used contraceptives more often than women who were not counseled (83.3% *v* 22.2%).

**Conclusion:**

A minority of young women with BC in Mexico use effective contraception methods during cancer treatment and receive contraceptive counseling. Informing all premenopausal patients with BC about effective use of contraception methods during treatment should be an essential aspect of the supportive care of young women.

## INTRODUCTION

Breast cancer (BC) represents the most frequent neoplasm and cause of cancer death in Mexican women.^1^ In Mexico, nearly 15% of women diagnosed with BC are age 40 years or younger, a higher proportion than in the United States and Canada each year (< 10%),^2-5^ and most of them will receive systemic therapy as part of BC management. Although BC treatment, especially chemotherapy and hormonal therapy, may have detrimental effects on ovarian function, it does not preclude the possibility of pregnancy during treatment. Therefore, contraceptive counseling and adherence have significant importance because these patients may have an unintended pregnancy.^6^

However, addressing the topic of contraception is complex for young women with BC because the currently available contraceptive methods are heterogeneous and nonhormonal options are limited.^7,8^ In addition, amenorrhea as a result of chemotherapy and hormonal therapy may lead patients and physicians to believe that treatment causes temporary infertility, and thus, that no contraception is needed during this time.^9^

Current studies from high-income countries show that use of effective contraceptive methods by patients with BC is suboptimal.^6,7,10^ However, there are no studies in the literature regarding contraceptive use in patients with BC in Mexico and other Latin American countries. The main aims of this study were to determine the rate of contraceptive use in young Mexican women with BC during cancer treatment, characterize their contraceptive preferences, and assess contraceptive counseling by Mexican physicians.

## PATIENTS AND METHODS

A cross-sectional study was conducted at the National Cancer Institute (Instituto Nacional de Cancerología [INCan]), a large tertiary health care facility in Mexico City. All women age 40 years or younger at BC diagnosis who had completed chemotherapy in the previous 5 years or who were currently receiving long-term treatment with hormonal therapy and/or trastuzumab were eligible to enroll. All suitable patients completed a self-administered 15-item paper survey with multiple-choice questions regarding contraceptive use and counseling. The survey was adapted from questions asked or variables in previous studies^7,11^ and was revised and approved by experts in the field (Data Supplement).

Contraceptive methods were classified according to the WHO’s tiers of effective contraception.^12^ Tier I is for long-lasting methods such as vasectomy and tubal ligation, hormonal implants, and copper and progesterone intrauterine devices. These methods are the most effective (failure rate, < 1% per year). Tier II is for methods with short duration, such as hormonal contraceptives in a variety of forms: pills, patches, intramuscular injections, and intravaginal rings (failure rate, 6% to 12% per year). Tier III is for barrier methods such as condoms and sponges (failure rate, 18% to 24% per year). Tier IV is for behavioral methods such as the calendar-based rhythm method and coitus interruptus (failure rate, > 24% per year). Contraceptive use was divided in three groups: effective (abstinence and tier I), noneffective (tiers II to IV), and no contraceptive use.

Study procedures were reviewed and approved by the INCan’s Institutional Review Board, and written consent was obtained from all participants. All women included in this study received a fact sheet with appropriate information on contraceptive use for patients with BC, and women with additional questions were referred to a gynecologic oncologist at our institution for additional information. Sociodemographic characteristics were obtained from medical records and the answered surveys. Clinical and pathologic variables were also obtained from medical records.

We determined associations using Fisher’s exact test and Pearson’s χ^2^ for categorical variables and Student *t* test for continuous parametric variables. A *P* value of < .05 was considered statistically significant. Univariable logistic regression assessed the association between contraceptive use and sociodemographic factors, stage at diagnosis, and treatment. Variables associated with univariable *P* values < .20 were evaluated in a multivariable logistic regression model using stepwise selection, and variables achieving significance at *P* < .05 were included in the final model. Statistical analyses were performed using SPSS v20.0 (SPSS, Chicago, IL).

## RESULTS

A total of 104 patients were eligible and all completed the survey. Median age at diagnosis was 34 years (range, 19 to 40 years), and 50.0% were age 35 to 40 years. Table 1 lists the patients’ sociodemographic characteristics. Briefly, most of them had one or more children, did not have a college degree, had a partnered relationship, and had stage II disease. Notably, almost 10% of the patients included in the study were stage IV at diagnosis, but optimal contraceptive use is important in patients receiving treatment regardless of clinical stage. Ninety-two patients (88.5%) received chemotherapy as part of their treatment, and 54 (58.7%) reported amenorrhea during treatment. Seventy patients (67.3%) received hormonal therapy, and 38 (54.3%) had amenorrhea during treatment.

**Table 1:**
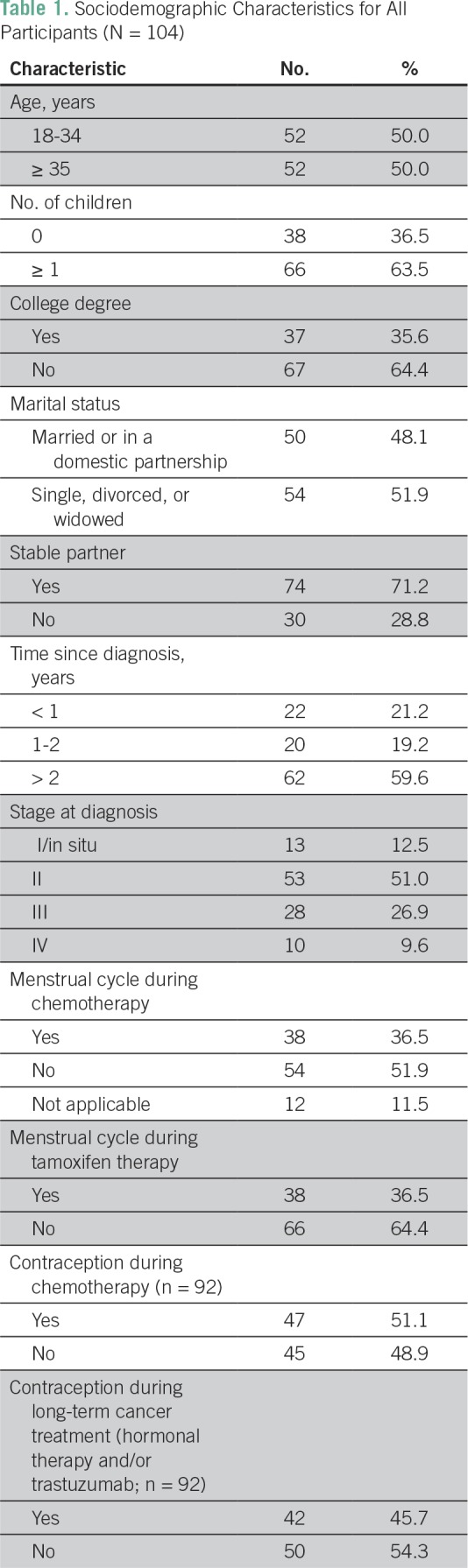
Sociodemographic Characteristics for All Participants (N = 104)

During chemotherapy, only 51.1% of patients (47 of 92) reported using a contraceptive method; barely 45.7% of patients undergoing treatment (42 of 92) used a contraceptive method throughout hormonal therapy and/or treatment with trastuzumab. Table 2 summarizes the reported choices of contraceptive use for patients receiving any type of cancer treatment and for those receiving long-term cancer therapy (hormonal treatment and/or trastuzumab). It is important to note that one patient used hormonal therapy as a contraceptive method. Notably, 49% of patients (51) were sexually active while receiving treatment, but only 76.5% used contraception during chemotherapy. Moreover, only 29.4% of patients (15) who reported sexual activity used an effective contraceptive method (tier I). Fortunately, despite reports of suboptimal use of effective contraceptive methods, there were no pregnancies during the study period.

**Table 2:**
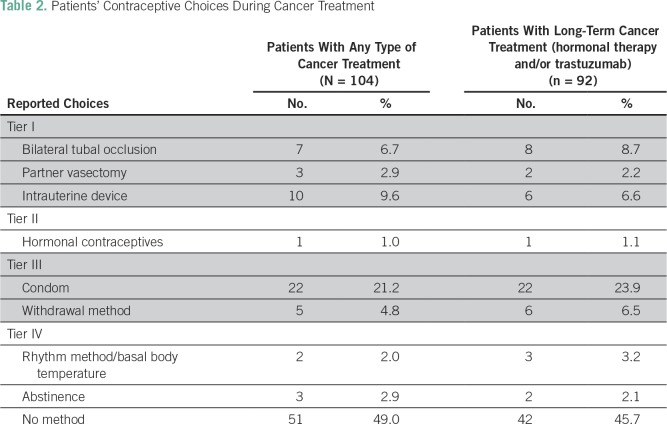
Patients’ Contraceptive Choices During Cancer Treatment

The associations between sociodemographic variables and effective contraceptive use during treatment are summarized in Table 3. Clinical stage, having a stable partner, not having children, and being sexually active during treatment were associated with contraceptive use. After multivariable analysis, only being sexually active remained significant (odds ratio, 21.714; 95% CI, 4.625 to 101.946; *P* < .001).

**Table 3:**
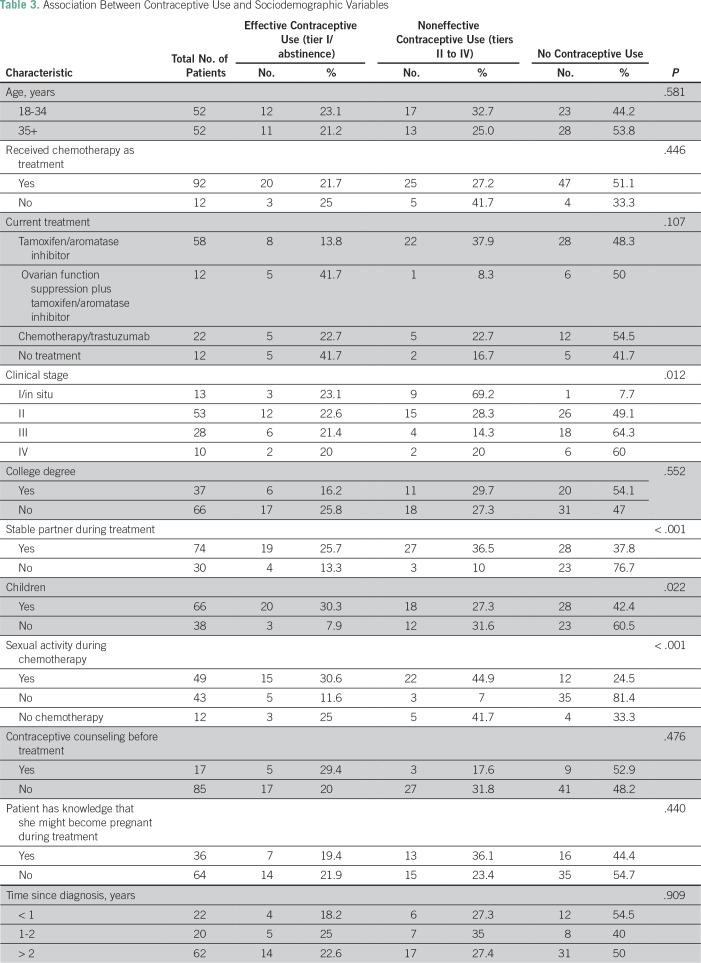
Association Between Contraceptive Use and Sociodemographic Variables

When asked about contraceptive counseling, only 16.7% of patients (17) recalled being advised by health care providers about using contraceptives during treatment, and only 34.6% of patients (36) knew about the possibility of becoming pregnant despite presenting with amenorrhea associated with BC treatment. Sixty-two percent of patients with BC (65) reported wanting to have more information about safe and effective contraceptive methods. Moreover, when only sexually active patients were analyzed (n = 51), contraceptive counseling was associated with effective contraceptive use (83.3% of counseled women used effective contraception *v* 22.2% of women who were not counseled; *P* = .011), but this did not remain statistically significant in the multivariable analysis.

## DISCUSSION

To the best of our knowledge, this is the first study describing contraceptive use in young women with BC in Mexico and Latin America. Our findings are similar to those in other BC populations in higher-resource settings as previously reported,^6,7,10^ with suboptimal use of effective contraception in these patients. These findings are particularly relevant because in Latin America, BC is diagnosed at a young age, and women younger than age 50 years represent approximately half the patients with BC.^5^

In sexually active patients with BC, use of contraceptive methods is comparable to that reported by the Instituto Nacional de Estadistica, Geografia e Informatica (INEGI [National Institute of Statistics, Geography and Informatics]) in Mexican women of the same age (75.6%).^13^ Although patients with BC should rely only on nonhormonal contraceptive methods, paradoxically, the use of such contraception is even lower in patients with BC (31.7%) compared with that in the general population (47.7%).^13^

In this study, only 29.4% of patients who reported sexual activity used an effective contraception method (tier I), the majority used noneffective contraceptive methods from tiers III and IV, and unexpectedly, one patient used hormonal contraception. The reasons for inappropriate selection of contraceptive methods in patients with BC, as also suggested by other authors,^14^ might include misinformation and lack of information on contraceptive options and risks, delayed discussions of contraceptive needs, limited guidance on family planning, lack of focus by health care professionals on reproductive health, and peers being trusted as a source of contraceptive information.

In this study, only being sexually active was statistically significantly associated with use of contraception. Others have also reported that higher education level is associated with effective contraceptive use, although we did not confirm those findings.^7^ Notably, when patients who reported sexual activity were analyzed exclusively, counseling was also an important factor for choosing an effective method. Thus, providing adequate advice had a positive impact on patients for their use of appropriate methods of contraception during treatment, regardless of sociodemographic characteristics.

Another important finding is that patients were unaware of the possibility of becoming pregnant during treatment for BC. This belief may be attributed to patients who present with amenorrhea during systemic therapy and who thus consider pregnancy unlikely. In our study, only 34.6% of patients knew about the potential for becoming pregnant during treatment. Although unintended pregnancy is a rare event, reported in approximately 1% to 3% of patients during treatment for BC,^6,10^ it should be discouraged mainly because of the risk of abortion and severe malformations.

Although it has been previously reported that giving contraceptive counseling has a positive impact on contraceptive use, only 16% to 60% of the physicians give advice about contraception to patients with BC.^7,15,16^ Regrettably, in our study, only 16.7% of our patients recalled being counseled by their physician about contraceptive use.

Our study has several limitations. Its cross-sectional design could lead to recall bias, and the small sample included only patients from one tertiary cancer care center in Mexico. It is likely that contraceptive use in other regions of Mexico or Latin America are even lower than that reported in our study, because INCan is one of the main referral cancer centers in our country. Despite the small number of patients included in our study, the findings are clinically relevant and should promote the immediate design and implementation of strategies to improve the use of effective contraception. Strategies might include educating patients, partners, and health care providers; providing easy access to reproductive health clinics in cancer centers or training health care personnel to provide contraceptive counseling; and reassuring that effective contraception should be used before and during treatment. The first steps being taken at INCan to promote education are the development and distribution of educational materials such as electronic and printed infographics regarding contraceptive use and effective methods (Data Supplement), and checklists for patients and providers that cover topics that must be discussed.^17^ In addition, patients who belong to the young women’s program are frequently reminded of contraceptive use during cancer treatment, and those with additional inquiries are referred to the two gynecologic oncologists at the BC unit.

Only a minority of young women with BC who are treated in a tertiary care center in Mexico use effective contraception methods during cancer treatment and receive contraceptive counseling. Even worse, patients who use less specialized centers may have lower use of effective contraception than that reported in our study. It is imperative to develop educational strategies to improve knowledge among physicians and patients about use of contraceptives and risk of pregnancy during BC treatment and to facilitate referrals to reproductive health specialists as needed. Informing all premenopausal patients about effective use of contraceptive methods during treatment should be an essential aspect of the supportive care of young women.

## References

[1] Knaul FM Nigenda G Lozano R et al Breast cancer in Mexico: A pressing priority Reprod Health Matters 16 113 123 2008 19027629 10.1016/S0968-8080(08)32414-8

[2] Brinton JT Barke LD Freivogel ME et al Breast cancer risk assessment in 64,659 women at a single high-volume mammography clinic Acad Radiol 19 95 99 2012 22054804 10.1016/j.acra.2011.09.003 PMC3261722

[3] Partridge AH Ruddy KJ Kennedy J et al Model program to improve care for a unique cancer population: Young women with breast cancer J Oncol Pract 8 e105 e110 2012 23277772 10.1200/JOP.2011.000501 PMC3439235

[4] Ali A Warner E pynk: Breast cancer program for young women Curr Oncol 20 e34 e39 2013 23443036 10.3747/co.20.1131 PMC3557339

[5] Villarreal-Garza C Aguila C Magallanes-Hoyos MC et al Breast cancer in young women in Latin America: An unmet, growing burden Oncologist 18 26 34 2013 24334479 10.1634/theoncologist.18-S2-26

[6] Güth U Huang DJ Bitzer J et al Unintended pregnancy during the first year after breast cancer diagnosis Eur J Contracept Reprod Health Care 21 290 294 2016 27227578 10.1080/13625187.2016.1180678

[7] Maslow BS Morse CB Schanne A et al Contraceptive use and the role of contraceptive counseling in reproductive-aged women with cancer Contraception 90 79 85 2014 24792148 10.1016/j.contraception.2014.03.002

[8] Güth U Huang DJ Bitzer J et al Contraception counseling for young breast cancer patients: A practical needs assessment and a survey among medical oncologists Breast 30 217 221 2016 26521069 10.1016/j.breast.2015.10.003

[9] Karaöz B Aksu H Küçük M A qualitative study of the information needs of premenopausal women with breast cancer in terms of contraception, sexuality, early menopause, and fertility Int J Gynaecol Obstet 109 118 120 2010 20152978 10.1016/j.ijgo.2009.11.027

[10] Johansen SL Lerma K Shaw KA Contraceptive counseling in reproductive-aged women treated for breast cancer at a tertiary care institution: A retrospective analysis Contraception 96 248 253 2017 28645785 10.1016/j.contraception.2017.06.004

[11] Cook-Andersen H Komrokian S DeMichele A et al Breast cancer patients have lower rates of contraception use Fertil Steril 96 S201 S202 2011

[12] Festin MP Kiarie J Solo J et al Moving towards the goals of FP2020: Classifying contraceptives Contraception 94 289 294 2016 27287693 10.1016/j.contraception.2016.05.015 PMC5032916

[13] INEGI Fecundidad mujeres mexicanas 2010. http://cuentame.inegi.org.mx/poblacion/discapacidad.aspx

[14] Mody SK Panelli DM Hulugalle A et al Contraception concerns, utilization and counseling needs of women with a history of breast cancer: A qualitative study Int J Womens Health 9 507 512 2017 28790868 10.2147/IJWH.S136120 PMC5531568

[15] Ramamurthy S Willis R Lee D Contraception in premenopausal breast cancer patients on tamoxifen: Have you advised? J Clin Oncol 29 2011 suppl; abstr e11558

[16] Crafton SM Lynch CD Cohn DE et al Reproductive counseling, contraception, and unplanned pregnancy in fertile women treated by gynecologic oncologists Gynecol Oncol Rep 19 22 26 2016 28018956 10.1016/j.gore.2016.11.006 PMC5173313

[17] Joven & Fuerte (2017). www.jovenyfuerte.com.mx,.

